# Standardization of Laboratory Test in the JPHC Study

**DOI:** 10.2188/jea.11.6sup_81

**Published:** 2007-11-30

**Authors:** Minoru Iida, Shinichi Sato, Masakazu Nakamura

**Affiliations:** 1 Osaka Medical Center for Cancer and CVD.; 2 Osaka Medical Center for Health Science and Promotion.

**Keywords:** CDC, CRMLN, accuracy, precision

## Abstract

The standardization committee has carried out standardization of 23 laboratories in the cohort area. They participated in the External Quality Control Survey by the Japan Medical Association. Most laboratories got A or B in evaluation criteria for most test items, but the results of AST, ALT and gamma-GTP were unsatisfactory. As for the lipid standardization, accuracy and precision of all 23 laboratories were satisfactory except for one. Close communication and collaborative study with reference laboratory improved the accuracy control.

## INTRODUCTION

Laboratory data which were sent from each study area should be standardized to compare each result. The standardization committee (Chairman: Dr. Iida) has carried out standardization of 23 laboratories in the cohort area. Osaka Medical Center for Cancer and CVD is a member of Cholesterol Reference Method Laboratory Network (CRMLN), and contributed to standardize lipid measurement in Japan for both epidemiology and clinical chemistry.

## METHODS OF STANDADIZATION

The subjects for standardization were 10 items that were measured at the health screening program in the local health centers by the Law of Promoting Health for Elderly. These included total protein, glucose, uric acid, creatinine, total cholesterol, AST (GOT), ALT (GPT), gamma-GTP, triacylglycerol, and HDL cholesterol. Accuracy control and standardization among each laboratory in the cohort area were performed by the Division of Mass Screening, Osaka Medical Center for Cancer and CVD.

Basically, External Quality Control Survey by the Japan Medical Association was employed. Accurate reference value was available for total cholesterol, HDL cholesterol and triacylglycerol by the CRMLN Lipid Standardization Program through Osaka Medical Center for Cancer and CVD.

External Quality Control Survey by the Japan Medical Association has been planed in every June, called for participation in July, and 4 samples were sent to laboratories in September. Each participant measured samples and reported the results to the Association in October. The results were evaluated according to the evaluation criteria (Tables 1a-c﻿﻿), and returned to each participant in the next February. We collected copies of these evaluation sheet from each laboratory to the Osaka Medical Center for Cancer and CVD, and evaluated.

**Table 1a.** Evaluation criteria for clinical laboratory test by Japan medical association. Total protein, uric acid, creatinine, total cholesterol (4 samples).

Evaluation criteria	Evaluation	Point
Within +/- 1 SD	A	2.5
+/- 1 SD to +/- 2 SD	B	2
+/- 2 SD to +/- 3 SD	C	1
over +/- 3 SD	D*	0
No participation	–	0

**Table 1b.** Evaluation criteria for clinical laboratory test by Japan medical association. AST, ALT, gamma-GTP (4 samples).

Evaluation criteria	Evaluation	Point
Within +/- 1 SD	A	3 (4)
+/- 1 SD to +/- 2 SD	B	2 (3)
+/- 2 SD to +/- 3 SD	C	1 (1)
over +/- 3 SD	D*	0 (0)
No participation	–	0 (0)

Point for ratio of measured value in parenthesis.

**Table 1c.** Evaluation criteria for clinical laboratory test by Japan medical association. Triacylglycerol, HDL cholesterol (2 samples).

Evaluation criteria	Evaluation	Point
Within +/- 1 SD	A	5
+/- 1 SD to +/- 2 SD	B	4
+/- 2 SD to +/- 3 SD	C	2
over +/- 3 SD	D*	0
No participation	–	0

Thirty sample sera for lipid analysis were sent to the laboratories in December or at the time of mass screening, according to the CRMLN Lipid Standardization Program by Osaka Medical Center for Cancer and CVD. Each laboratory should randomly select 3 samples for one day, and repeat duplicate measurement for 10 consecutive days. We collected the data and evaluated the results according to the criteria (Table 2). If the results were acceptable, laboratories could receive Certification for Clinical Laboratory issuid by CDC/CRMLN.

**Table 2.** Criteria for acceptable performance of the CDC-NHLBI lipid standardization program.

Item	Concentration level (mg/dl)	Precision (standard deviation)	Accuracy (bias from RV)
Total cholesterol	100-149	4.00	0.03 RV
	>150	0.03 RV	0.03 RV
LDL cholesterol	50.0-99.9	2.00	2..0
	<40.0	3.00	3.0
HDL cholesterol	<40.0	2.50	0.10 RV
	40-59.9	3.00	0.10 RV
	>=60.0	3.50	0.10 RV
Triacylglycerol	0-88	7	9
	89-176	8	10
	177-220	10	11
	>=221	0.05 RV	0.05

RV; reference value, from Criteria for Acceptable Performance of the CDC-NHLBI Lipid Standardization Program

## RESULTS OF STANDADIZATION AMONG LABORATORIES

The participation of the 28th External Quality Control Survey by the Japan Medical Association in 1994 by each laboratory were shown in Table 3. Sixteen of 24 laboratories sent us the results. Participation status to the CRMLN Lipid Standardization Program was shown in Table 4. Twenty-three laboratories participated in the second study which was carried out during January and February in 1995.

**Table 3.** Participation in the external quality control survey by the Japan medical association.

Health center	Lab name	1991	1992	1993	1996
		25th	26th	27th	28th
Yokote	Hiraga General Hospital		◎	◎	
	Akita General	◎	◎	◎	

Ninohe	Iwate	◎	◎	◎	◎
	SRL Hachioji Lab	◎	◎	◎	

Kasama	Ibaragi General Health Institute	◎	◎	◎	◎

Katsushika					

Saku	Nagano Health Control Center	◎	◎		◎
	Tsuchiya Enterprise				◎
	Chikuma Hospital				
	Koumi Red Cross Hospital				

Kashiwazaki	Kashiwazaki Medical	◎	◎	◎	◎
	Ojiya General Hospital	◎	◎		◎

Suita	National Cardiovascular Center	◎	◎	◎	◎
	Osaka University Research Institute for Microbial Diseases	◎	◎	◎	◎

Tosayamada	Tosayamada	◎		◎	◎
	Kochi General Health Institute	◎	◎	◎	

Arikawa	Kamigoto Hospital			◎	◎
	Arikawa Hospital			◎	◎
	Narao Hospital			◎	◎
	Odika Clinic			◎	◎
	Fukue Public Health Center			◎	◎

Ishikawa	Okinawa General Health Institute	◎	◎	◎	◎
	Ishikawa Public Health Center				

Miyako	Miyako Public Health Center			◎	◎

	Total:	12	12	17	16

**Table 4.** Participation in the lipid standardization program.

Health center	Lab name	1st study	2nd study
		1994 Jan.-Feb.	1995 Jan.-Feb.
Yokote	Hiraga General Hospital	◎	◎
	Akita Prefectural General Health Association	◎	◎

Ninohe	Iwate	◎	◎
	SRL Hachioji Laboratories	◎	

Kasama	Ibaraki Health Service Association	◎	◎

Katsushika		◎	◎

Saku	Health Care Center Nagano Prefectural Federation of Agricultural Cooperatives for Health and Welfare	◎	◎
	Tsuchiya Enterprise		◎
	Chikuma Hospital		◎
	Koumi Red Cross Hospital		◎

Kashiwazaki	Kashiwazaki Kariwa Medical Association	◎	◎
	Kashiwazaki Medical Center		
	Ojiya General Hospital	◎	◎

Suita	National Cardiovascular Center	◎	◎
	The Reserch Foundation for Microbial	◎	◎
	Disease of Osaka University		

Tosayamada	Tosayamada Public Health Center	◎	◎
	Kochi Health Service Association	◎	◎

Arikawa	Kamigoto Hospital	◎	◎
	Arikawa Hospital	◎	◎
	Narao Hospital	◎	◎
	Ojika Clinic	◎	◎
	Fukue Public Health Center	◎	◎

Ishikawa	Okinawa General Health Service Association	◎	◎
	Ishikawa Public Health Center	◎	◎

Miyako	Miyako Public Health Center	◎	◎

	Total:	21	23

**Figure 1.**
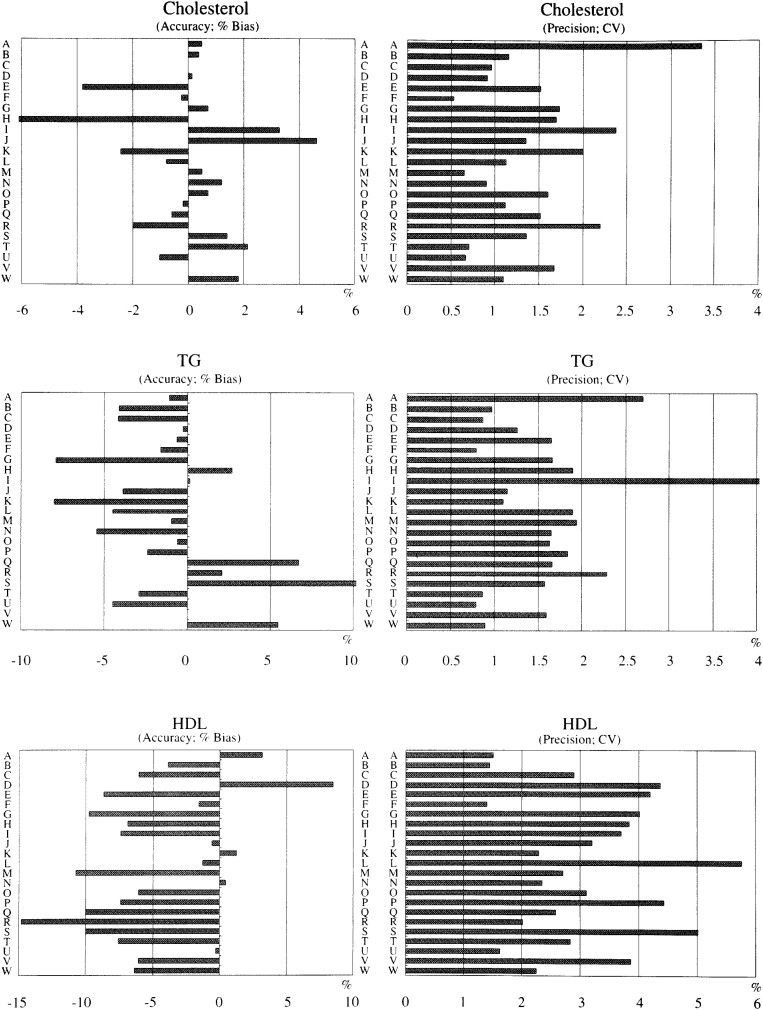
Accuracy (left column) and precision (right column) of cholesterol, triacylglycerol and HDL cholesterol of 23 laboratories.

Evaluation of 16 laboratories was summarized in Table 5. Most laboratories got A or B in evaluation criteria for most test items, but the results of AST, ALT and gamma-GTP failed to be C or D category in many laboratories. As for the 2^nd^ Lipid Standardization Program, accuracy and precision of 23 laboratories are shown in Figure 1. Accuracy of cholesterol measurement was satisfied in 19 out of 23 and precision satisfied in all laboratories. Triacylglycerol measurement was satisfied in both accuracy and precision by all laboratories except one. The same was for HDL cholesterol measurement.

**Table 5.** Evaluation of laboratory test from 16 laboratories.

Test item	Participating laboratories	Criteria	Sample no.							

			1	(%)	2	(%)	3	(%)	4	(%)
glucose	16	A	11	68.8	14	87.5	13	81.3	13	81.3
		B	5	31.3	2	12.5	3	18.8	2	12.5
		C	0	0.0	0	0.0	0	0.0	1	6.3
		D	0	0.0	0	0.0	0	0.0	0	0.0

uric acid	15	A	10	66.7	13	86.7	13	86.7	10	66.7
		B	4	26.7	2	13.3	2	13.3	3	20.0
		C	1	6.7	0	0.0	0	0.0	2	13.3
		D	0	0.0	0	0.0	0	0.0	0	0.0

creatinine	16	A	13	81.3	12	75.0	14	87.5	12	75.0
		B	2	12.5	4	25.0	1	6.3	2	12.5
		C	0	0.0	0	0.0	0	0.0	1	6.3
		D	1	6.3	0	0.0	1	6.3	1	6.3

Total-cholesterol	16	A	13	81.3	12	75.0	13	81.3	13	81.3
		B	3	18.8	4	25.0	8	18.8	2	12.5
		C	0	0.0	0	0.0	0	0.0	1	6.3
		D	0	0.0	0	0.0	0	0.0	0	0.0

AST	16	A	10	62.5	9	56.3	9	56.3	7	43.8
		B	2	12.5	5	31.3	4	25.0	6	37.5
		C	3	18.8	1	6.3	1	6.3	2	12.5
		D	1	6.3	1	6.3	2	12.5	1	6.3

ALT	16	A	9	56.3	7	43.8	10	62.5	7	43.8
		B	4	25.0	5	31.3	2	12.5	6	37.5
		C	2	12.5	4	25.0	4	25.0	3	18.8
		D	1	6.3	0	0.0	0	0.0	0	0.0

*γ*-GTP	16	A	10	62.5	8	50.0	9	56.3	8	50.0
		B	4	25.0	4	25.0	5	31.3	5	31.3
		C	0	0.0	3	18.8	0	0.0	2	12.5
		D	1	6.3	0	0.0	1	6.3	0	0.0

Total-protein	14	A	8	57.1	10	71.4				
		B	6	42.9	4	28.6				
		C	0	0.0	0	0.0				
		D	0	0.0	0	0.0				

Triacyl-glycerol	16	A	12	75.0	12	75.0				
		B	4	25.0	3	18.8				
		C	0	0.0	1	6.3				
		D	0	0.0	0	0.0				

HDL-cholesterol	16	A	10	62.5	11	68.8				
		B	5	31.3	5	31.3				
		C	1	6.3	0	0.0				
		D	0	0.0	0	0.0				

## PROBLEMS OF FUTURE RESOLUTION

From the results of External Quality Control Survey by the Japan Medical Association, AST, ALT and gamma-GTP measurement showed the problem in accuracy control. These should be improved. On the other hand, the results of lipid standardization program were satisfactory. This is the result of our close contact in the cohort study since the project had started. The difficult problem for quality control on the laboratories was that the health screening data were indirectly obtained from laboratories that were nominated by the local city, town or village which should carry out the health screening for the residents by the law. At the beginning of this study, direct communication between reference laboratory and each laboratories was not established, but it has been improved recently, as shown by the result of lipid standardization. The quality control shall be repeated every year, so the laboratory data should become satisfactorily comparable in the future.
